# Primary Malignant Melanoma of Renal Pelvis with Extensive Clear Cell Change

**DOI:** 10.7759/cureus.583

**Published:** 2016-04-21

**Authors:** George Liapis, Helen Sarlanis, Elpida Poulaki, Konstandinos Stravodimos, Olga Riccioni, Andreas C Lazaris

**Affiliations:** 1 1st Department of Pathology, School of Medicine, National and Kapodistrian University of Athens; 2 Medical School, La Sapienza University of Rome

**Keywords:** clear cell, kidney, melanoma, pelvis, primary

## Abstract

Our presentation illustrates a rare case of primary renal pelvis malignant melanoma in a 35-year-old man. The diagnosis of malignant melanoma was based on immunophenotype and the detection of intracellular melanin pigment. The renal origin was proven by the presence of scattered melanocytes within the urothelium of the pelvis. The tumor exhibited extensive clear cell change that closely mimics clear cell renal cell carcinoma. The patient’s clinical history did not disclose any signs of previous melanocytic skin or mucosa lesions. Differential diagnosis includes tumors capable of synthesizing melanin or expressing melanocytic markers.

## Introduction

Primary malignant melanomas (MM) of the genitourinary tract constitute less than 1% of all MMs [1]. Primary melanoma of the kidney is an extremely rare type of tumor; only four cases of primary renal MM in adulthood have been described so far in the reviewed literature [2-5]. On the contrary, the kidney is often affected by metastatic melanoma mainly in the form of multiple cortical micrometastases [6] while renal pelvis involvement is usually secondary to a primary lesion of the posterior trunk [7]. 

Herein, we report a case of a 35-year-old man presenting with MM of the renal pelvis with extensive clear cell histological appearance. There was no clinical history of MM and no skin or mucosal lesions were found in the physical examination and endoscopy performed before and after diagnosis.

## Case presentation

A 35-year-old man was admitted to Laiko General Hospital due to macroscopic hematuria. A computed tomography (CT) scan revealed a mass of tumor that measured 2.5 cm in the largest diameter. The tumor was located in the pelvis of the right kidney. A nodular lesion that measured 3 cm in diameter was also found in the VII segment of the right liver lobe. The patient’s previous clinical history was unremarkable, and his familial history did not disclose any relevant information. A right nephrectomy was performed, and a liver biopsy was obtained via surgical procedure.

A tumor measuring 2.5 cm × 2.2 cm × 2 cm was found in the renal pelvis protruding in the calyx and the peripelvic fat. The renal pelvis was partially obstructed. In cut sections, the tumor showed a solid texture with a whitish color. The rest of the kidney did not show any macroscopic lesions. The whole tumor as well as a liver biopsy specimen 1 cm in length were processed for histological examination according to the standard protocol. Formalin-fixed and paraffin-embedded tissue sections were prepared for light microscopy examination.

Immunohistochemical assays with antibodies for the detection of HMB-45, Melan-A, CD-10, WT-1, vimentin, pan-cytokeratin, SMA, chromogranin, synaptophysin, CD56 (DAKO, Glostrup, Denmark), and S-100 (THERMO SCIENTIFIC, Waltham, MA, USA) epitopes were performed under standard protocols in tumor tissue sections.

**Figure 1:**
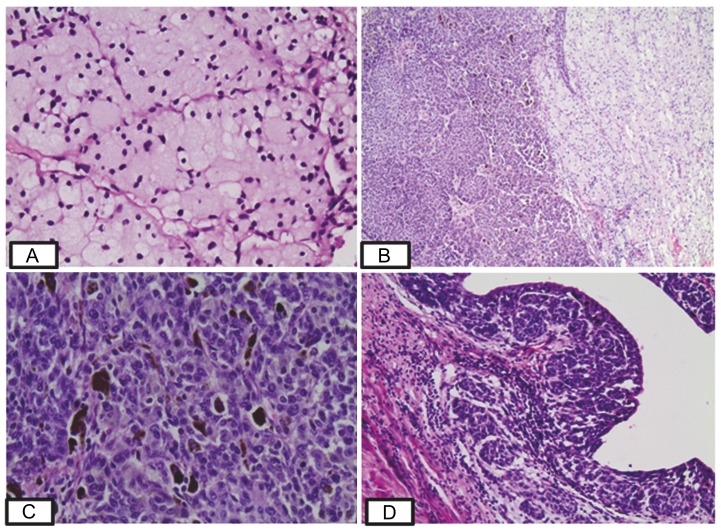
H&E staining of the pelvic tumor. A: Neoplastic cells with clear cytoplasm arranged in nests separated by thin fibrous septa (400×). B: Sheets of basophilic round cells in abrupt transition to the clear cell component (100×). C: Basophilic round cells, higher magnification. Melanin pigment is demonstrated (200×). D: Nests of tumor cells within and beneath the urothelium having a resemblance with “nevoid melanoma” (200×).

The examined pelvic tumor exhibited marked heterogeneity on histological grounds. The predominant pattern was composed of sheets or nests of clear cells separated by thin fibrous septa. Cells exhibited micro-vacuolated cytoplasm and round nuclei with indistinct nucleoli (Figure 1A). A secondary tumor component was also identified, characterized by confluent nests and sheets of round basophilic cells with chromatin-dense nuclei in an abrupt transition to the clear cell element (Figure 1B). Mitoses were numerous in the latter tumor area. Intranuclear inclusions were occasionally noted while melanin pigment deposition was also recognizable in a few areas (Figure 1C). Nests of tumor cells were also encountered within and beneath the urothelium with a resemblance to nevoid melanoma (Figure 1D).

Biopsy material revealed metastatic infiltration of the liver tissue by the clear cell component of the renal tumor. 

**Figure 2:**
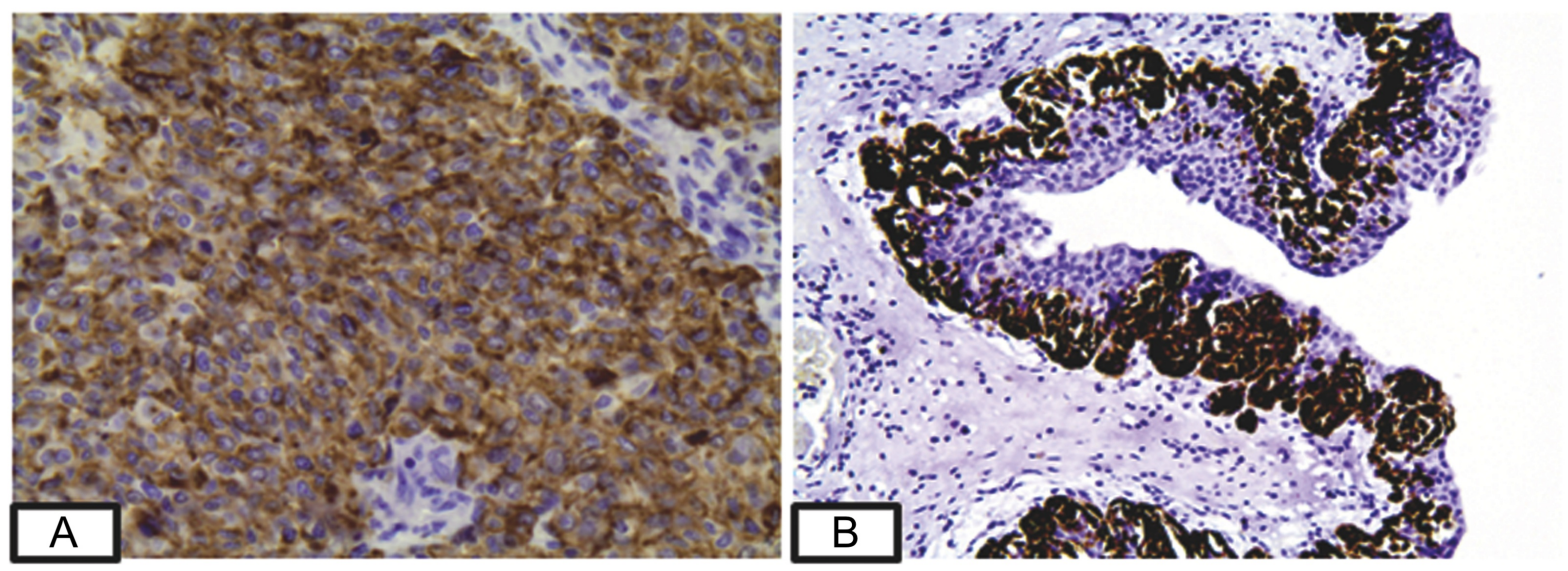
HMB-45 staining of the pelvic tumor. A: Diffuse expression of HMB-45 in tumor cells (400×). B: Presence of melanocytes within the urothelium highlighted by HMB-45 (200×).

A positive reaction was seen for all melanocytic markers (HMB-45 (Figure 2A), S-100, Melan-A, and cytoplasmic reaction for WT-1) both in renal tumor and liver lesion. CD10, a marker of renal clear cell carcinoma that can also be expressed in melanoma [8], was positive in our case. Vimentin was also positive, while pan-cytokeratin, SMA, and the neuroendocrine markers chromogranin, synaptophysin, and CD56, were negative. Surprisingly, melanocytic markers revealed the presence of melanocytes within the urothelium, even in areas considerably distant from the main tumor (Figure 2B).

The final histological diagnosis was primary renal pelvis MM. The diagnosis was based on the detection of intracellular melanin pigment and the tumor immunophenotype while the renal origin was supported by the presence of melanocytes within the urothelium of the pelvis and by the exclusion of any primary melanocytic lesions of the skin or mucosal tissues. The liver nodule was considered metastatic.

After six months and under chemotherapy, a metastatic mass in the spleen was diagnosed via CT scan examination.

### Materials and methods

Upon contact with the patient and approval of the Bioethics Committee of the Clinicolaboratory Sector, School of Medicine, The National and Kapodistrian University of Athens, the present case material was retrieved from the archive of the First Department of Pathology so that this report is made.

## Discussion

We present the 5th case of primary renal MM in adulthood. Table 1 presents information regarding the cases reported to date, including patient presenting symptoms and medical history, imaging and examinations results, surgical procedure, macroscopic findings, histological and immunohistochemical data, and follow-up.

**Table 1:** Clinicopathologic features of primary renal MM cases reported in adulthood to date.

	Frasier et al. 1988 [3]	Tajima et al. 1997 [4]	Bayazit et al. 2002 [5]	Tasdemir et al. 2011 [2]	Liapis et al. 2016
Patient	37-year-old white man	74-year-old Japanese woman	37-year-old male	67-year-old man	35-year-old man
Presenting symptoms	Gross hematuria and right flank pain	Pollakisuria	Localized, dull, right lumbar pain	Right lumbar pain	Macroscopic hematuria
Investigations					
Abdominal CT	Rounded, soft tissue mass confined to the right renal pelvis; no evidence of visceral metastasis	Right renal mass with irregular internal density; no evidence of visceral metastasis	7-cm heterogenic mass in the right kidney; 3-cm lesion within the paracaval area extending towards the adrenal gland	Right renal mass with irregular internal density; no evidence of visceral metastasis	2.5-cm tumor mass in the pelvis of the right kidney; 3-cm nodular lesion in the VII segment of the right liver lobe
Physical examination	Right flank and costovertebral angle tenderness	Unremarkable findings; no skin lesions	7-8-cm mobile mass in the right upper quadrant of the abdomen; no skin lesion	Unremarkable findings; no skin lesions	Unremarkable findings; no skin lesions
Urinalysis	Full field of red blood cells	Slight microscopic hematuria		Slight microscopic hematuria	(Macroscopic hematuria as presenting symptom)
Surgical procedure	Nephroureterectomy	Right radical nephrectomy	Radical nephrectomy with adrenalectomy, Paracaval and interaortacaval lymph node dissection	Right radical nephrectomy	Right nephrectomy, Liver biopsy obtained during surgical procedure
Macroscopic findings	Tumor of about 5.0 x 4.0 x 3.0 cm, feeling the entire renal pelvis, without extending into the renal parenchyma	Solid tumor of about 3-3.5 cm, showing a dark brown color		Mass of about 4.5 cm, showing areas of focal hemorrhage	Tumor of about 2.5x2.2x2 cm in the renal pelvis, protruding in the calyx and the peripelvic fat
Evaluation of the surgical specimins					
Histology	Organized nest of markedly pleomorphic cells with abundant eosinophilic cytoplasm and large hyperchromatic nuclei; isolated areas with fine dust-like golden pigment within the cytoplasm of tumor cells. Tumor invasion into the smooth muscle of the renal pelvis	Extensive proliferation of mainly clear cells; brown pigment deposits in the cytoplasm of the tumor cells	Tumor composed of epitheloid cells showing nodular architecture; prominent melanin pigmentation in the cytoplasm of the tumor cells and stroma. Tumor invasion into the adjacent fatty tissue. Metastases in the paracaval lymph nodes	Tumor cells with large eosinophilic cytoplasm and large nucleus. Tumor thrombus in the lumen of the renal vein	Tumor marked heterogeneity; predominant pattern composed of nests of clear cells with micro-vacuolated cytoplasm and round nuclei; melanin pigment deposition in few areas. Nests of tumor cells within and beneath the urothelium. Metastatic infiltration of liver tissue
Immunohistochemistry	Positivity for: S-100 protein, and stains for melanoma antigens. Negativity for: iron stains, and keratine stains	Positivity for: Fontana-Masson stain, Vimentin, S-100 protein, Neuron-specific enolase, and HMB-45	Positivity for: HMB-45, and Fontana-Masson stain	Positivity for: HMB-45, and S-100 protein	Positivity for: HMB-45, S-100, Melan-A, WT-1, CD10, and Vimentin. Negativivity for: Pan-cytokeratin, SMA, Chromogranin, Synaptophysin, and CD56
Treatment	Bacuìillus Calmette-Guerin and allogenic melanoma cell vaccination protocol as adjuvant immunotherapy regimen	Human lympfoblastoidinterferon-alfa therapy; interruption after 1 month (due to patient's general fatigue and appetite loss)	Fotemustine and interferon-alfa 2b therapy for 3 months (in another institution)	Human lympfoblastoidinterferon-alfa therapy; interruption after 1 month (due to patient's general fatigue and appetite loss)	Chemotherapy
Follow-up	1 year after initial presentation: recurrence at surgical incision treated by wide excision. 22-month follow-up: no evidence of disease	2 years and 3 months after surgery: no evidence of residual disease	1 year later (on his second admission): neoplastic lesions in the right nephrectomy space, in the liver right lobe and in the lung, detected via CT. Urooncology follow-up care (he refused chemotherapy) until he died (because of respiratory problems)		6-month followup: metastatic mass in the spleen detected via CT

Most MMs of the kidney are metastatic, thus, a careful examination of histological features in combination with the evaluation of clinical data and CT scans is demanded. Differential diagnosis includes tumors capable of producing melanin and/or expressing melanocytic markers such as melanin-pigmented renal cell carcinoma [9], melanin-producing perivascular epithelioid cell tumor (PEComa) [10] and X11 translocation renal cell carcinoma [11]. Moreover, renal pelvis melanoma may show clear cell appearance mimicking conventional clear cell carcinoma [12]. Melanin-pigmented renal cell carcinoma is negative for melanocytic markers while the cytoplasm of tumor cells is clear. Furthermore, positivity of CD10 and vimentin in tumor cells does not aid in differential diagnosis between these two distinct entities. PEComa is negative for S-100 and positive for SMA. X11 translocation renal cell carcinoma may express Melan-A, but the histological features are different [11]. In addition, tumors showing a wide histological spectrum (such as Wilms tumor and clear cell sarcoma) may enter the differential diagnosis. However, tumor cell immunophenotype and melanin pigment deposition are opposed to both diagnoses.  

The origin of the tumor is still obscure because the kidney is devoid of melanocytes [13]. Regarding the pathogenesis of urogenital melanoma in general, it is speculated that migrating precursor melanocytes from the neural crest may be the source of origin [14]. The established criteria for defining primary melanoma of the bladder [15,16] may be also applied for the identification of the primary origin of renal pelvis melanoma. The following criteria are suggested: no previous cutaneous lesion history or any evidence of regressed cutaneous melanoma; no evidence of visceral melanoma in other parts; the pattern of recurrence should be consistent with the primary lesion; and the urothelium should contain atypical melanocytes or *in situ* melanoma at the vicinity of melanoma mass.​

## Conclusions

In conclusion, our presentation illustrates a rare case of renal pelvis melanoma. It poses malignant melanoma as a potential differential diagnosis for renal tumors capable of synthesizing melanin or expressing melanocytic markers, especially considering the new entities that have been described such as X11 translocation renal cell carcinoma. The extensive clear cell histological appearance, closely mimics conventional renal clear cell carcinoma. A unique feature not mentioned in previous studies, is the presence of scattered melanocytes within the urothelium.
